# Constructing Machine Tool Foundations Using an LMP Alloy

**DOI:** 10.3390/ma13071649

**Published:** 2020-04-02

**Authors:** Yi Zhang, Wanlu Chen, Suqin Dou, Panpan Li, Hai Gu, Ren-E Dong

**Affiliations:** 1 School of Mechanical Engineering, Xijing University, Xi’an 710123, China; chenwanlu@xijing.stu.cn (W.C.); dousuqin@xijing.edu.cn (S.D.); lipanpan@xijing.stu.cn (P.L.); dongrene@xijing.edu.cn (R.-E.D.); 2 Jiangsu Key Laboratory of 3D Printing Equipment and Application Technology, Nantong Institute of Technology, Nantong 226002, China; guhaint@ntit.edu.cn

**Keywords:** LMP alloy, machine tool foundation, grouting, hot bath method, alloy recycling

## Abstract

Currently, the construction of machine tool foundations is a complicated and lengthy procedure with a limited flexibility. In this paper, we present a novel system for constructing machine tool foundations that replaces the need for concrete or concrete-polymer hybrids with a low melting point (LMP) alloy. The system uses a hot bath method to maintain the LMP alloy grouting in liquid form. A fixing device is used to control the embedded depth and positional accuracy of the foundation bolt assembly. The grouting material is injected into the foundation pit by a filling device. This can be extracted from the foundation pit in a later stage with the aid of a recycling device, enabling new machine tool foundations to be manufactured by reusing the LMP alloy grouting material. A prototype was built to test the proposed design. The results show that the system can construct machine tool foundations in a single application, without the delays associated with concrete-based construction, lowering both the economic and environmental cost.

## 1. Introduction

Machine tool foundations are mainly used to fix machine tool lathe beds, to bear the load imposed by machine tools and workpieces, to absorb the vibration generated during machining, and to isolate machines from external vibration sources. As noted by both Novotny et al. [1] and Yang et al. [2], the construction quality has a direct impact on the working accuracy and service life of a machine tool.

**Figure 1:**
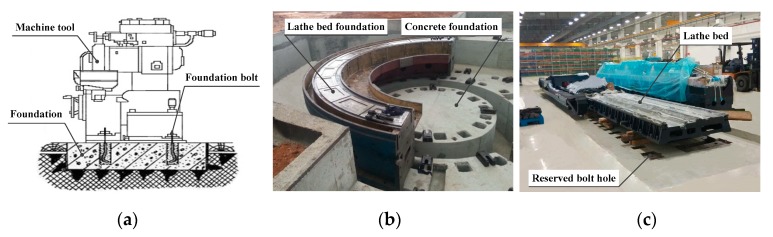
Conventional approach to constructing machine tool foundations: (**a**) Structure of a machine tool foundation; (**b**) a concrete machine tool foundation; (**c**) a lathe bed waiting for secondary grouting.

The basic structure of a machine tool foundation is shown in Figure 1. The material usually used to construct machine tool foundations is concrete [2], though over the years, various adaptations to this have been proposed, such as ferrocement [3,4], resin concrete [5,6], epoxy resin concrete [7,8], and hybrid polymer concrete [9]. When concrete or concrete-based materials are used as the grouting to construct foundations for heavy or precision machine tools, the process typically involves the handling of a large volume of construction material that is poured in several stages to provide the basic foundation, followed by primary and secondary grouting. Various other processes are also necessary, including waterproofing, treatments against corrosion, anti-seismic protection, and recurrent maintenance. This not only makes the construction process complicated, but also places stringent quality requirements on the engineering procedure [10,11], consumes considerable amounts of time, and makes concrete-based construction expensive [12]. Traditional machine tool foundations also require ongoing maintenance and lack versatility. Therefore, if different types of machine tools are installed on an original foundation, differences in structure make it necessary to remove the previous foundation and construct a new one from scratch, which further increases the construction costs.

**Figure 2:**
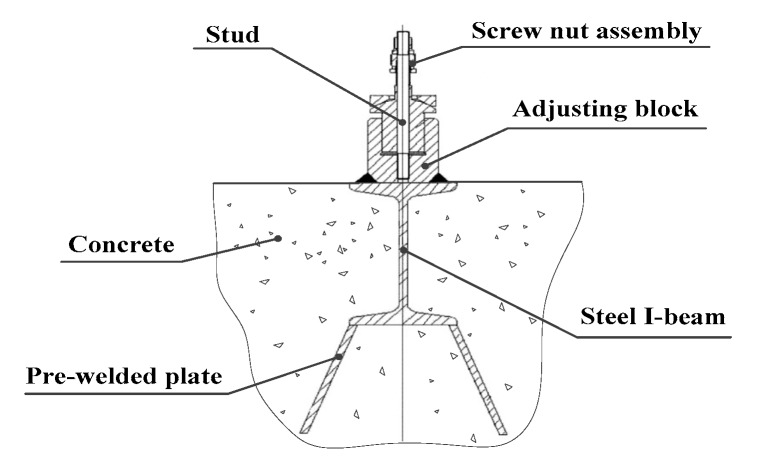
A single-step approach to constructing machine tool foundations [12].

**Figure 3:**
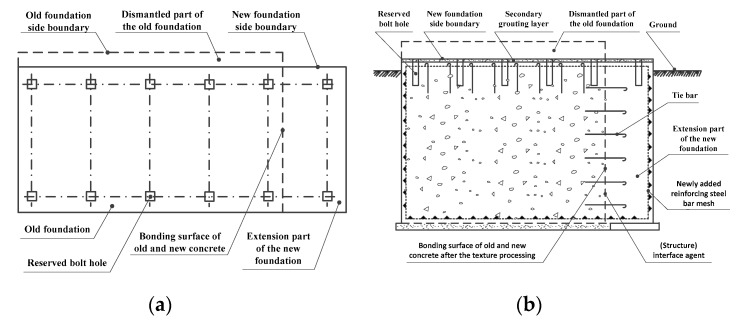
A composite foundation composed of old and new concrete [13]. (**a**) Top view; (**b**) longitudinal section.

In view of the problems noted above, a lot of research has been devoted to finding a solution. Wang et al. [12] developed a single-step forming method for constructing machine tool foundations. In this method, a structural body is prepared by welding together steel I-beams and pre-welded plates. This is then embedded in the foundation. After this, the machine tool is fixed and locked onto adjustable blocks with a nut assembly, as shown in Figure 2. This method eliminates the need for phased construction, solves the hidden quality hazards of having I-beams only embedded at shallow depths, and does not require reserved holes for foundation bolts. This saves the time and construction cost involved in primary grouting. However, when the adjustable blocks were fixed onto the steel I-beams via groove welding, large welding deformation was produced. It also proved difficult to arrive at a reasonable layout for the steel I-beams, which made it hard to complete pre-adjustment and installation of the lathe bed. To meet certain design specifications, Xu et al. [13] constructed a composite foundation containing new and old concrete by taking an original power machine foundation as the main body, removing redundant parts and material, and adding other missing elements. In order to satisfy the performance requirements of the machine, it was necessary to add a textured surface to the interface between the old and new concrete, spray on an interface agent, and add tie bars to reinforce the new foundation, as shown in Figure 3. This shortened the construction period, saved concrete and steel bars, and reduced construction costs. However, a prerequisite for this kind of procedure is a considerable structural similarity between the old and new foundations, which greatly limits its scope of application.

In the approach proposed in this paper, the above problems are solved by means of a novel machine tool construction system that is based on the innovative use of new low melting point (LMP) alloys to replace the use of concrete and concrete-polymer hybrids. Although there has been some prior exploration of using alloys in place of concrete because of the scope they offer for recycling [14], this is the first paper to propose using them as the principal grouting for machine tool foundations. An LMP alloy typically has a melting point of 120–180 °C and the system detailed in this paper makes use of this characteristic so that different types of machine tool foundation can be constructed in the same foundation pit. An LMP alloy is a binary or multicomponent alloy with a melting point below 232 °C. It most commonly consists of the elements Bismuth (Bi), Lead (Pb), Tin (Sn), Cadmium (Cd), Indium (In), or Aluminum (Al). An LMP alloy has a good fluidity and thermal conductivity after heating, a low expansion and shrinkage rate after solidification, and a good stability [15]. These characteristics enable it to meet the varied requirements of machine tool foundation construction for grouting and it is easy to recycle and reuse. In particular, an LMP alloy offers the prospect of a single pouring of the grouting, obviating the problem of having to allow curing time between phases. This is further facilitated here through the use of an innovative ‘hot bath’ method for directly applying LMP alloys in situ. With an LMP alloy, there is no need to provide waterproofing or anti-corrosion treatments and it has a low maintenance cost. Therefore, unlike the solution proposed by Xu et al. [13], the system being proposed here does not involve the recurrent expense of removing prior foundations and having to prepare a bonding surface and interface. Considering this, a key advantage of the approach being put forward here is that the use of the LMP alloy can ensure that machine tool foundations are both structurally flexible and open to rapid recycling. A further advantage of the proposed system is that, rather than providing a foundation assembly where the steel fixings are configured offsite, here, a construction frame allows for flexible onsite configuration of the machine tool pedestal. Therefore, the approach being advocated here can provide a specific system that is able to comprehensively cover all aspects of the construction process, thereby speeding up the construction time, significantly improving the construction efficiency, and lowering the construction cost. It also provides a specific application case for the processing of alloys that has broader implications for future green construction processes.

The rest of the paper provides a basic overview of the proposed machine tool construction system and how it works, before going into greater detail about the specific subsystems and their constituent parts. This is followed by a description of the testing of the system’s viability using a small prototype and a discussion of the test results and their implications. Therefore, the system is tracked from its specific design to a trial application. The paper concludes by considering the potential value of adopting this approach for the construction of machine tool foundations in the future.

## 2. System Structure and Function

**Figure 4:**
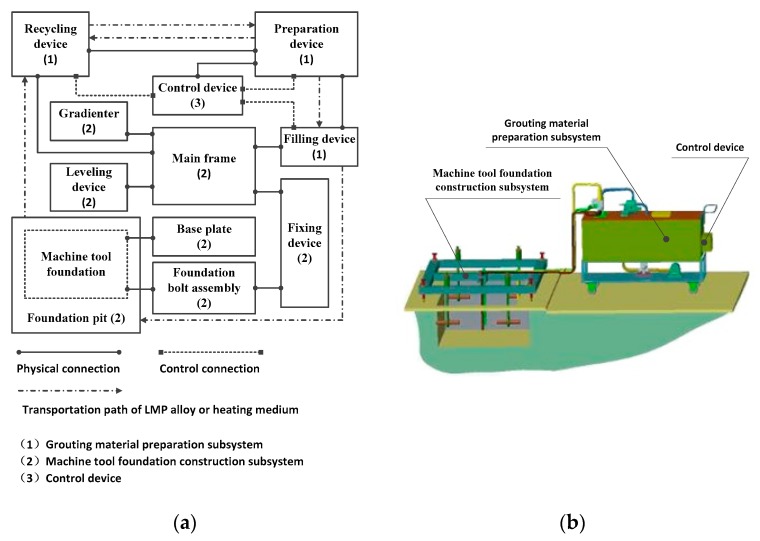
Structural layout and 3D model of the system. (**a**) Basic structural layout; (**b**) 3D model.

In this paper, a novel system is presented for the construction of machine tool foundations that is based upon an LMP alloy rather than concrete. The system is based upon a complex ecology of interrelated elements that are organized around three core subsystems: a grouting material preparation subsystem; a machine tool foundation construction subsystem; and a controller. Its basic structural layout and a 3D model are shown in Figure 4. Sequentially, the grouting material preparation subsystem has the task of preparing the grouting material so that it is ready to be used and the machine tool foundation construction subsystem then actually constructs the foundation. There is an additional recycling element within the material preparation subsystem that has the task of recovering excess material or grouting already present in the foundation pit, so that it may be re-used. The controller handles the interaction between the subsystems by managing the flow of material through various channels and conduits.

The grouting material preparation subsystem is mainly composed of a preparation device, a filling device, and a recycling device [16]. The preparation device uses a hot bath approach to prepare the liquid LMP alloy grouting material. This is then transferred to the machine tool foundation construction subsystem via the filling device. When a prior machine tool foundation is being removed or reconfigured, the recycling device can be used to extract discarded liquid LMP alloy grouting from the foundation pit and transport it to the preparation device for reuse. The recycling device is also used to transport the heating medium to the preparation device at room temperature.

The machine tool foundation construction subsystem includes a main frame, a fixing device, foundation bolt assemblies, a leveling device, a gradienter, and a base plate [17]. Although it is effectively a pre-existing element, it also makes sense to incorporate the foundation pit within this subsystem because it is implicated within the overall process in several different ways. The fixing device, leveling device, and gradienter are installed on the main frame and welded to a steel channel. The fixing device is used to position and fix the foundation bolt assemblies. During the grouting construction process, the base level of the main frame is adjusted to a horizontal position by the leveling device with the help of the gradienter to ensure that the screw axis of the foundation bolt is always perpendicular to the horizontal plane. The liquid alloy grouting produced by the grouting material preparation subsystem is transported to the foundation pit by the filling device for pouring it into the machine tool foundation. After the grouting material has cooled and solidified and its temperature has dropped to room temperature, any remaining heating medium in the foundation pit needs to be cleared out, and the base plate can then be positioned over the foundation bolt screws to cover the machine tool foundation. As the base plate is a load-bearing plate, it can dissipate the dynamic and static loads imposed by the machine tool and processing parts that are mounted on it into the whole upper face of the machine tool foundation, thus improving the foundation’s overall load-bearing capacity.

The control device uses an STC89C52 single chip microcomputer [18] as its core and has various pre-set parameters based upon the characteristics of the materials being used and input control instructions relating to the capacity of the system and its operation. Information collected by a liquid level sensor and a temperature sensor in the grouting material preparation subsystem is combined to control the operation of a solenoid valve, a pump, and a heater in the subsystem. In this way, it manages the preparation, filling, and recycling of the LMP alloy.

## 3. Detailed Design of the System

### 3.1. Grouting Material Preparation Subsystem

**Figure 5:**
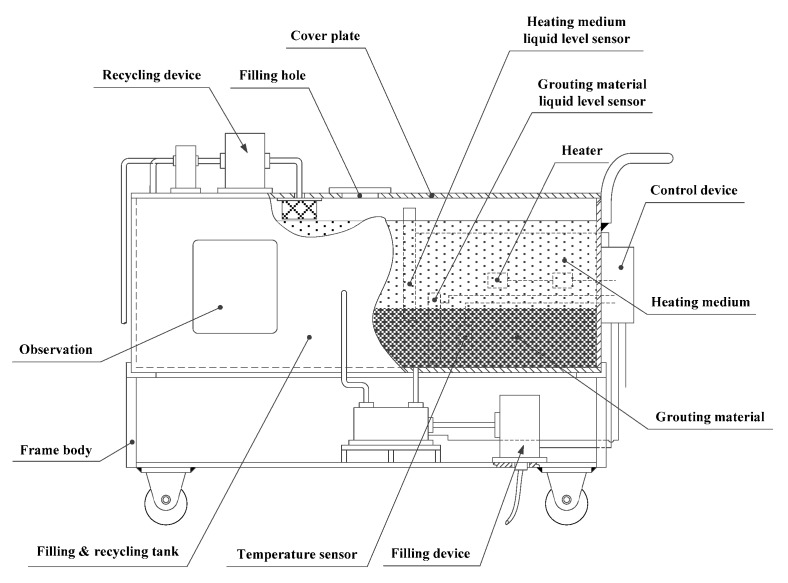
Grouting material preparation subsystem.

The grouting material preparation subsystem is used to both prepare and recycle the liquid alloy grouting. Its basic structure is shown in Figure 5.

#### 3.1.1. Preparation Device

The preparation device is installed on the body frame and is mainly composed of the filling and recycling tank, the heater, the grouting level sensor, the heating medium level sensor, and a temperature sensor.

The filling and recycling tank makes up the bulk of the preparation device. An NGEL650A nano-based soft felt thermal insulating layer (Shenzhen Furang Energy Saving Technology Co., Ltd., Shenzhen, China) is bonded to the outer wall of the tank and has a thermal conductivity at room temperature of only 0.003–0.012 w/(k·m). Having a good level of hydrophobicity helps to reduce the energy consumption when preparing the grouting material. A polytetrafluoroethylene (PTFE) film with a high temperature resistance and self-cleaning properties is sprayed on the inner wall of the tank to prevent the alloy grouting material from adhering to the wall.

The liquid LMP alloy grouting material is prepared using a hot bath method. Basically, this involves a heater heating a heating medium that is circulated around the grouting material until it has melted. When the melting point of the alloy is below 100 °C, water can be used as the hot bath medium. If the melting point is between 100 and 232 °C, methyl silicone oil can be used as the hot bath medium instead. The heater is used to raise the temperature of the heating medium until it reaches the melting point of the alloy grouting. It is made up of several 304 stainless steel double-ended U-shaped electric heater pipes in parallel. The number of pipes is determined by various constraints, including how much grouting material needs to be produced within a set time, the heat absorbed by the alloys, the nature of the heating medium, the thermal properties of the filling and recycling tank, and the amount of heat dissipated by the nano-based soft felt insulating layer.

The formula for calculating the heat absorbed by the heating medium, $Q_{m}$, is shown in Equation (1):

(1) $$Q_{m}=C_{m}S_{bc}H_{m}\rho_{m}\left(T_{ms}-T_{mi}\right),$$

where, $C_{m}$ is the specific heat capacity of the heating medium, $S_{bc}$ is the cross-sectional area of the filling and recycling tank, $H_{m}$ is the depth of the heating medium, $\rho_{m}$ is the density of the heating medium, $T_{ms}$. is the set temperature (working temperature) of the heating medium, and $T_{mi}$ is the heating medium’s initial temperature.

The formula for calculating the heat absorbed by the alloy, $Q_{a}$, is shown in Equation (2):

(2) $$Q_{a}=C_{as}m_{a}\left(T_{o}-T_{ai}\right)+q_{a}m_{a}+C_{al}m_{a}\left(T_{as}-T_{o}\right),$$

where, $C_{as}$ is the specific heat capacity of the solid alloy; $m_{a}$ is the mass of the alloy; $T_{o}$ is the melting temperature of the alloy; $T_{ai}$ is the initial temperature of the solid alloy, and $T_{ai}=T_{mi}$; $q_{a}$ is the melting heat of the alloy; $C_{al}$ is the specific heat capacity of the liquid alloy; $T_{as}$ is the set temperature of the liquid alloy, and $T_{as}=T_{ms}$; and $T_{as}>T_{o}$.

The formula for calculating the heat absorbed by the filling and recycling tank, $Q_{b}$, is shown in Equation (3):

(3) $$Q_{b}=C_{b}m_{b}\left(T_{bs}-T_{bi}\right),$$

where, $C_{b}$ represents the specific heat capacity of the tank material; $m_{b}$ represents the mass of the tank; $T_{bi}$ represents the initial temperature of the tank, and $T_{bi}=T_{mi}$; and $T_{bs}$ represents the set temperature of the tank, and $T_{bs}=T_{ms}$.

The formula for calculating the average heat loss of the nano-based soft felt thermal insulation layer, $Q_{f}$, is shown in Equation (4):

(4) $$Q_{f}=\frac{1}{2}S_{f}q_{lr}t_{sh},$$

where, $S_{f}$ represents the surface area of the insulation layer, $q_{lr}$ represents the rate of heat loss for the insulation layer when the temperature of the heating medium is set at $T_{ms}$, and $t_{sh}$ represents the time taken to heat the alloy from $T_{ai}$ to $T_{as}$.

The heat loss of the surface of the heating medium in the filling and recycling tank can be neglected if it is considered to be a closed container. The overall designed allowance for the impact of the constraints is 20%. On the basis of the above, the number of heating pipes, n, can be calculated as follows:

(5) $$n=ROUNDUP\left(\frac{1.2\left(Q_{m}+Q_{a}+Q_{b}+Q_{f}\right)}{P_{ht}t_{sh}},0\right),$$

where, $P_{ht}$ is the power of the heating pipe.

A mixture of technological and physical resources are provided to ensure that the grouting material and heating medium can be effectively monitored. An observation window, temperature sensor, and liquid level sensors for the heating medium and grouting material are installed on the side wall of the filling and recycling tank. The observation window enables the working state of the filling and recycling tank to be observed. By means of these various mechanisms, an operator can keep a close eye on how much heating medium and grouting material is currently in the tank and, if necessary, step in to control the filling and heating process. The type, function, and parameters of the three sensors are shown in Table 1.

As the temperature of the alloy grouting material does not need to go above 232 °C, a highly-sensitive and reliable copper-constantan thermocouple was selected as the temperature sensor. The temperature sensor transmits the overall liquid alloy temperature to the control device in real time. When the temperature of the alloy reaches the set value, $T_{as}$, the control device uses a Proportion-Integral-Differential (PID) algorithm to control the heater [19] and make sure that the liquid alloy remains at a constant temperature the whole time.

**Figure 6:**
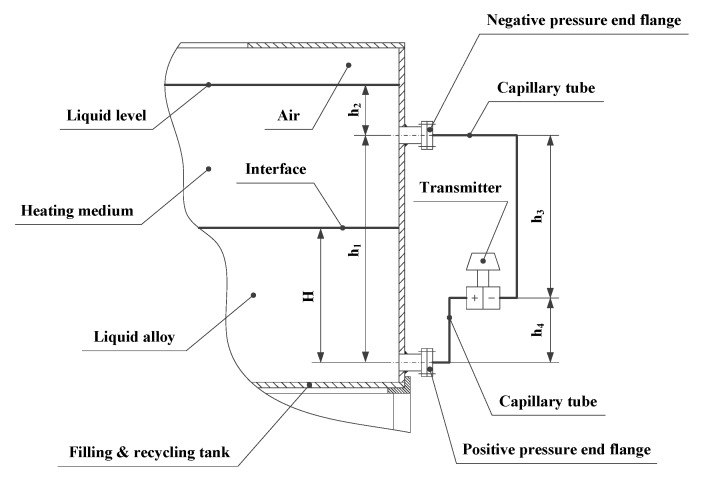
Differential pressure level gauge with double flanges.

A differential pressure liquid level meter with double flanges was adopted as the grouting material liquid level sensor. This measures the distance between the liquid alloy and the heating medium relative to the axis of a flange at the positive pressure end of the meter. This can be thought of as measuring the level of the interface. The meter is composed of a transmitter, capillary, pressure transfer medium, positive pressure end flange, and negative pressure end flange. The position at which each component is installed is shown in Figure 6.

As the interface is between two kinds of high-temperature liquids, the pressure transfer medium filling the two capillary tubes at the positive and negative pressure ends of the transmitter was chosen to be silicone oil. This can withstand temperatures of −15–250 °C. The range of the pressure measurement was an important consideration when selecting the differential pressure liquid level meter. It can be obtained by calculating the zero point and the measuring range of the transmitter, which involves adopting the following procedure.

The static pressure of the transmitter’s positive pressure chamber, $P_{+}$, can be calculated as follows:

(6) $$P_{+}=\rho_{a}gH+\rho_{m}g\left(h_{1}+h_{2}-H\right)+P_{0}-\rho_{s}gh_{4},$$

where, $\rho_{a}$ is the density of the liquid alloy, g is the gravitational acceleration, H is the interface level, $h_{1}$ is the distance between the axes of the flanges at the positive and negative pressure ends, $h_{2}$ is the distance between the level of the heating medium and axis of the flange at the positive pressure end, $P_{0}$ is the atmospheric pressure, $\rho_{s}$ is the density of the silicone oil in the capillary tubes, and $h_{4}$ is the vertical distance between the positive pressure chamber and the axis of the flange at the positive pressure end.

The static pressure of the transmitter’s negative pressure chamber, $P_{-}$, can be calculated as follows:

(7) $$P_{-}=\rho_{m}gh_{2}+P_{0}+\rho_{s}gh_{3},$$

where, $h_{3}$ represents the vertical distance between the negative pressure chamber and the axis of the flange at the negative pressure end.

Therefore, the differential pressure value between the positive and negative pressure chambers of the transmitter, ΔP, can be calculated by using Equation (8):

(8) $$\Delta P=P_{+}-P_{-}=\left(\rho_{a}-\rho_{m}\right)gH+\left(\rho_{m}-\rho_{s}\right)gh_{1}.$$

According to Equation (8), when H=0, the zero point of the transmitter, $\Delta P_{0}$, can be obtained as follows:

(9) $$\Delta P_{0}=\left(\rho_{m}-\rho_{s}\right)gh_{1}.$$

According to Equation (8), when $H=h_{1}$, the measuring range of the transmitter, $\Delta P_{r}$, can be calculated as follows:

(10) $$\Delta P_{r}=\left(\rho_{m}-\rho_{s}\right)gh_{1}.$$

As $\rho_{a}>\rho_{s}$ and $\rho_{a}>\rho_{m}$, the measurement range of the differential pressure liquid level meter with double flanges, $R_{p}$, can be calculated by using Equation (11):

(11) $$\left(\rho_{m}-\rho_{s}\right)gh_{1}\leq R_{p}\leq \left(\rho_{a}-\rho_{s}\right)gh_{1}.$$

The control device sets the upper and lower limit values for the level of the interface. When the grouting liquid level sensor indicates that the liquid has reached its upper limit, the control device issues an alert, which notifies the operator to stop adding solid alloy through the filling hole in the cover plate. When the measured value hits its lower limit, the control device also sends out an alert, so that more solid alloy can be added. This control method can firmly guarantee the continuous production of liquid alloy in a filling and recycling tank of a limited capacity, so that this does not constrain the pouring of the machine tool foundation.

It is not only the level of the grouting that needs monitoring, but also the level of the heating medium, as this, too, is expended during the filling process. Therefore, this is accomplished in the grouting material preparation subsystem by means of another differential pressure liquid level meter, this time with a single flange, but used in the same way. This assesses the real-time liquid level of the heating medium in the filling and recycling tank. The level of the heating medium is also controlled within upper and lower limits. The solid alloy and normal temperature heating medium are first added to the subsystem from an external source. As soon as the liquid level sensor for the heating medium reaches its lower limit, the control device sets the heater to work. When the level reaches its upper limit, the control device sends a signal to stop the addition of heating medium to the recycling device, so as to prevent it overflowing. During the process of preparing the liquid alloy, the control device ensures that the recycling device continues to fill the tank with the heating medium until the liquid’s level rises to its upper limit. The heating medium in the tank slowly reduces to its lower limit through various losses, at which point, the control device ensures that the filling begins again.

#### 3.1.2. Filling Device and Recycling Device

**Figure 7:**
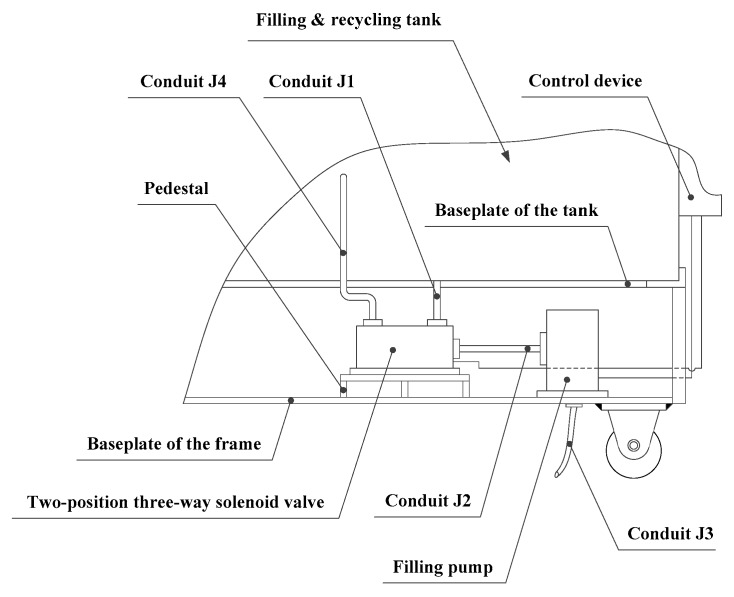
Filling device.

The principal structure for the filling device is installed on the baseplate of the frame. It incorporates the filling pump, a two-position three-way solenoid valve, and a pedestal, as shown in Figure 7. The outlet of the filling pump is connected to conduit J3. Conduit J2 connects the inlet of the filling pump to the outlet of the two-position three-way solenoid valve. The two-position three-way solenoid valve is fixed on the pedestal and has two inlets attached to it. Conduit J1 is attached to one of these inlets and passes through the baseplate of the tank to be connected to the liquid alloy grouting in the recycling tank. Conduit J4, which is connected to the other inlet, passes through the side wall of the recycling tank and is connected to the heating medium.

**Figure 8:**
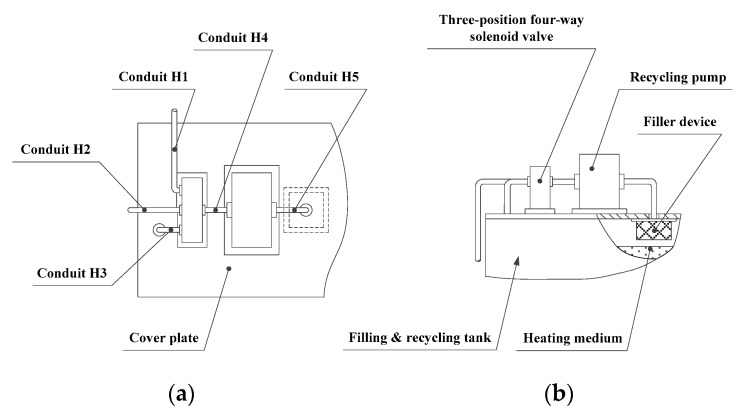
Recycling device. (**a**) Top view; (**b**) front view.

The recycling device principally consists of a recycling pump, a three-position four-way solenoid valve, a filtering device, and several conduits. Its basic structure is shown in Figure 8. The filtering device is fixed under the cover plate on the inner side of the filling and recycling tank. One end of conduit H5 is connected to the outlet of the recycling pump and the other end is connected to the filtering device. The three-position four-way solenoid valve has three inlets and one outlet. Conduits H1, H2, and H3 are connected to the three inlets. Conduit H1 is connected to the room temperature source of the external heating medium. Conduit H2 is connected to the machine tool foundation construction subsystem and conduit H3 is inserted into the heating medium reservoir in the filling and recycling tank, via the cover plate. One end of conduit H4 is connected to the outlet of the three-position four-way solenoid valve, and the other end is connected to the inlet of the recycling pump. When material for recycling needs to be extracted from the foundation pit, this is driven by the recycling pump via conduit H2.

To prevent the transportation channel from being blocked by any residual alloy grouting after cooling and solidification, the channels for both the filling device and the recycling device are designed with a self-cleaning function. After the filling or recycling of the liquid alloy grouting is completed, the high-temperature heating medium is extracted from the filling and recycling tank by using the filling or recycling pump, respectively. In this way, the grouting transportation channels are always flushed through.

Operation of the filling and recycling pumps and the solenoids and various valves is controlled through the control device. The functions of the filling device and recycling device are summarized in Table 2.

### 3.2. Machine Tool Foundation Construction Subsystem

**Figure 9:**
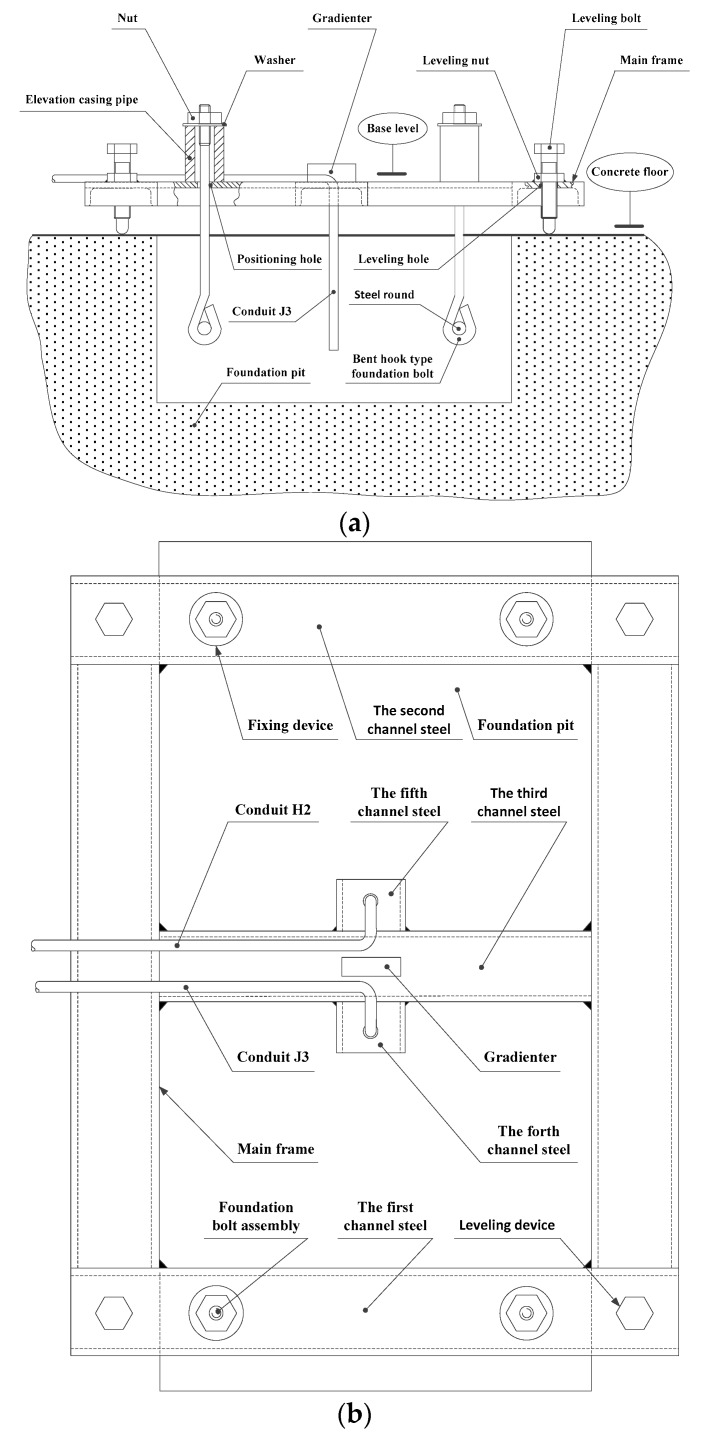
Machine tool foundation construction subsystem. (**a**) Front view; (**b**) top view.

The reliability and positional accuracy of the embedded foundation bolts have a direct impact on the quality of installation for machine tools. Different types of foundation bolts are anchored in different ways. If we take a block foundation that is level with the ground as an example, a bent hook-type foundation bolt can be embedded and anchored through a one-step process. At the time of pouring the grouting material, the foundation bolt is directly embedded, as shown in the structural overview in Figure 9.

The foundation bolt assembly is composed of a bent hook-type foundation bolt and a steel round, which is used to fix the machine tool to the lathe bed. The steel round passes through the bent hook of the foundation bolt horizontally. This prevents the bolt from rotating and improves its resistance to being pulled out once embedded.

The fixing device includes an elevation casing pipe, a nut, and a washer. These are used to locate the foundation bolt assembly. The number required equals the number of foundation bolt assemblies required for the machine tool installation. The screw of the foundation bolt assembly passes through a positioning hole in the main frame, from bottom to top, and is then suspended on the elevation casing pipe by the nut and washer. The correct elevation of the foundation bolt can be achieved by adjusting the axial dimension of the elevation casing pipe. If the diameter of the bolt is d, its embedded depth is not less than 20 d [20]. The position, size, and required accuracy of the fixing device on the main frame will need to be determined according to the structural parameters of the machine tool pedestal. These parameters are, in turn, derived from the given specifications for the specific machine tool.

Four leveling devices, consisting of leveling nuts and bolts, are installed in the first and second steel channels of the main frame, where a leveling nut is spot-welded onto the main frame’s base plane. The end of the leveling bolt is a hemispherical structure, which passes through the leveling nut and the leveling hole in the main frame. This presses on the concrete floor to a specified degree to directly support the main frame above the foundation pit. The four leveling bolts can be rotated separately, driven by the leveling nut, to perform a leveling operation with reference to the plane of the main frame. A gradienter installed in the third steel channel is used to determine the accuracy of the leveling.

Conduit J3 is connected to the outlet of the filling pump and passes through a hole in the fourth steel channel, before extending into the foundation pit to deliver the liquid alloy grouting or high-temperature heating medium. When the alloy grouting is added, the outlet of conduit J3 has to be higher than the level of the liquid alloy grouting, otherwise the conduit will be bonded to the foundation when the alloy solidifies. Conduit H2 passes through a hole in the fifth steel channel and is used in tandem with the recycling pump to extract any discarded alloy grouting or cooled heating medium from the foundation pit.

The foundation pit itself is used to hold the poured machine tool foundation. Although, in reality, existing foundation pits would currently be filled with more traditional grouting, such as concrete, to illustrate the proposed system’s full range of functionality, it is assumed here that the foundation pit will either be empty or populated with previously applied LMP alloy. In order to prevent the injected liquid alloy grouting and heating medium from penetrating beyond the foundation pit and causing environmental pollution, concrete should still be used in any case for the first construction of a foundation pit so as to form an outer casing. This will then remain in place for future instances of recycling and reconstruction.

The distance between the horizontal section edge of the foundation pit and the edge of the machine tool pedestal should be no less than 100 mm and the distance between the horizontal section edge of the foundation pit and the axis of the foundation bolts should be no less than 4 d [20]. The upper surface of the machine tool foundation is formed under the combined action of gravity and the surface tension of the liquid alloy grouting material, together with the gravitational impact of the heating medium, so it is effectively self-leveling. Its surface roughness and evenness of level meet the general requirements of machine tool installation, so it is no longer necessary to undertake additional leveling by adjusting the iron pad or other devices. To provide a block foundation that is level with the ground and a foundation pit of a particular depth, the influence of factors such as the machine tool foundation and base plate thickness needs to be considered. This can be calculated according to the following:

(12) $$D_{fp}=\frac{L_{mf}}{2W_{mf}}\sqrt[{3}]{\frac{G_{m}+G_{mp}+G_{p}}{W_{mf}E_{ag}y_{ad}}}+T_{p}$$

where, $D_{fp}$ represents the depth of the foundation pit, $L_{mf}$ represents the length of the machine tool foundation, $W_{mf}$ represents the width of the machine tool foundation, $G_{mt}$ represents the weight of the machine tool, $G_{mp}$ represents the maximum weight of a workpiece, $G_{p}$ represents the weight of the base plate, $E_{ag}$ represents the modulus of the alloy grouting material, $y_{ad}$ represents the allowable deviation in the foundation’s construction, and $T_{p}$ represents the thickness of the base plate.

### 3.3. Control Device

**Figure 10:**
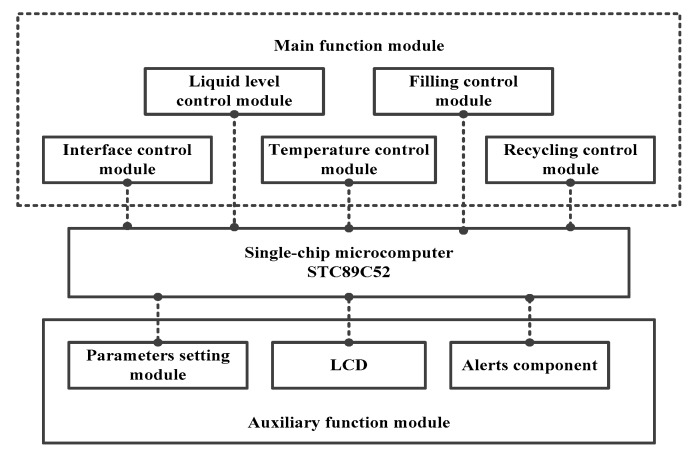
Block diagram of the control device and its functions.

The control device consists of five main function modules and three auxiliary function modules, as shown in Figure 10.

The parameter setting auxiliary module inputs the setting temperature of the liquid alloy, $T_{as}$; the upper limit value, $T_{ip(max)}$; and lower limit value, $T_{ip(min)}$, of the interface level; the upper limit value, $T_{llp(max)}$; and the lower limit value, $T_{llp(max)}$, of the grouting liquid level into an STC89C52 single chip microcomputer. An LCD (Liquid Crystal Display) then displays the various input values synchronously.

**Figure 11:**
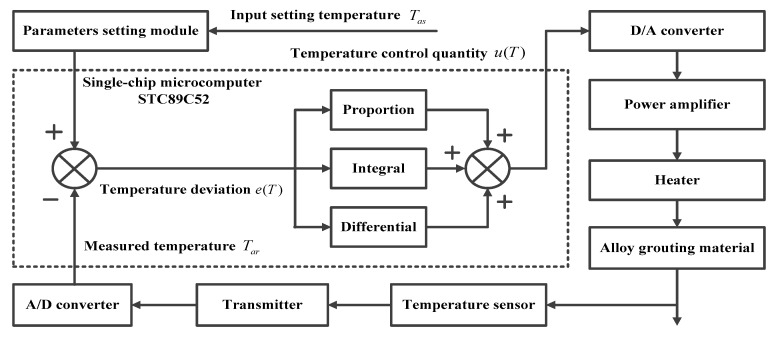
Working principle of the temperature control module.

As previously noted, the temperature control module uses a PID algorithm to control the alloy grouting preparation process. Its working principle is shown in Figure 11.

The temperature sensor collects the real-time temperature signal of the alloy grouting. The transmitter modulates this temperature signal into a DC (Direct Current) analog signal at 4–20 mA, which is transmitted to an A/D (Analogue/Digital) converter. The STC89C52 single-chip microcomputer (Hongjing Technology Limited, Hong Kong, China) compares the measured temperature, $T_{ar}$ (that is, the digital signal generated by the A/D converter), with the set temperature, $T_{as}$, to establish the temperature deviation, e(T). A linear combination of establishing the proportion, integrating, and then establishing the differential is carried out to define the temperature adjustment value, u(T). The calculations required for this are shown in Equations (13) and (14):

(13) $$e\left(T\right)=T_{ar}-T_{as}$$

(14) $$u\left(T\right)=K_{P}\left(e\left(T\right)+\frac{1}{T_{I}}\int e\left(T\right)dt+T_{D}\frac{de\left(T\right)}{dt}\right)$$

where, $K_{P}$ is the proportional coefficient, $T_{I}$ is the integral time constant, and $T_{D}$ is the differential time constant.

The temperature adjustment value, u(T), is processed using digital-analog conversion and power amplification. The output of this process is then used to control the operation of the heater, so that the temperature of the alloy grouting is kept constant after having reached the initial $T_{as}$. The LCD displays the set temperature, $T_{as}$, and the measured temperature, $T_{ar}$, in real time.

The interface control module controls the process of adding the solid alloy grouting by adjusting the quantity. After starting the grouting material preparation subsystem, the single-chip microcomputer compares the interface level data, H, collected by the grouting material liquid level sensor with the stored value for the upper limit of the interface level, $T_{ip(max)}$, in real time. When $H=T_{ip(max)}$, the microcomputer generates a high-level signal and triggers the alert component. The addition of solid alloy grouting to the recycling tank is then halted. During the process of injecting liquid alloy grouting into the foundation pit and pouring the machine tool foundation, when $H=T_{ip(min)}$, the interface level control module issues another alert when it is necessary to indicate that solid alloy grouting now needs to be added to the recycling tank again. This ensures a continuous supply of grouting material to the preparation subsystem. The liquid level control module works in a similar way to the interface level control module to control the addition of heating medium.

At the construction site, when the temperature of the liquid alloy grouting reaches the set temperature, $T_{as}$, the filling control module sends a control signal to the two-position three-way solenoid valve of the filling device to connect Channel (3), as shown in Table 3. It then starts the filling pump to fill the high-temperature heating medium to preheat the foundation pit. Subsequently, the solenoid valve is controlled to connect Channel (1) so that the liquid alloy grouting is added to the foundation pit. After the filling work is finished, Channel (2) is connected and any liquid alloy grouting material remaining in the filling channel is cleared away by using the high-temperature heating medium in the filling and recycling tank.

The recycling control module fulfils four kinds of function in relation to the recycling device, by controlling the recycling pump and the three-position four-way solenoid valve. When the liquid alloy grouting material is first being prepared, the recycling control module controls the three-position four-way solenoid valves so as to connect Channel (4). It then starts the recycling pump to import the heating medium at its ambient temperature into the recycling tank. When recycling discarded alloy grouting material from the machine tool foundation, Channel (3) is connected first of all and the high-temperature heating medium is extracted from the filling and recycling tank by the filling pump so that the alloy grouting material can be liquefied. Then, Channel (5) is connected so that the recycling pump can extract the heating medium and liquefied alloy grouting material from the foundation pit. After the recycling is finished, Channel (6) is connected by controlling the three-position four-way solenoid valves and the recycling pump is re-started to extract the high-temperature heating medium from the filling and recycling tank so as to clear the recycling channel of liquid alloy grouting material.

## 4. Prototype and Testing

**Figure 12:**
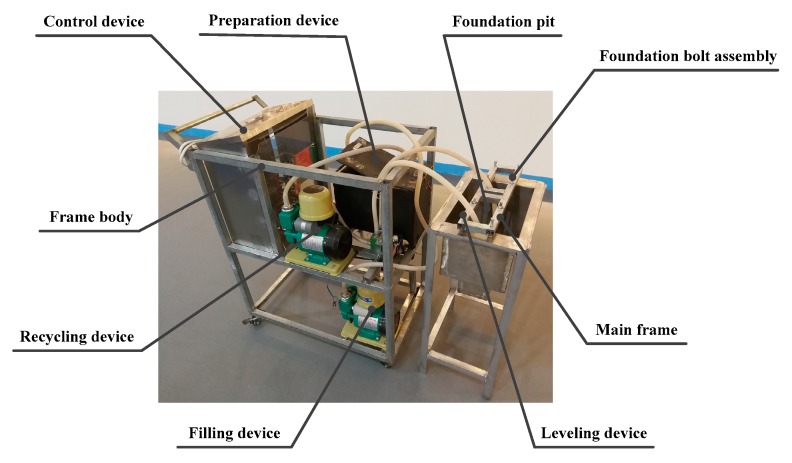
The basic prototype.

A small experimental prototype was built in the laboratory to verify the feasibility and effectiveness of the LMP alloy machine tool foundation construction system (see Figure 12). The prototype included the preparation device, filling device, recycling device, frame body, main frame, foundation bolt assembly, leveling device, foundation pit, and control device. The specifications of some of the main materials that make up this prototype are shown in Table 4.

A Bi-Sn-In alloy was selected as the grouting material for testing. The main components of a Bi-Sn-In alloy are 61.8% Bi, 10.0% Sn, and 28.2% In. Its solid phase temperature is 80.5 °C, its liquid phase temperature is 91.8 °C, and its melting range is 11.3 °C.

### 4.1. Preparation of the Alloy Grouting Material

As a first step, the cover plate of the preparation device was opened and solid Bi-Sn-In alloy grouting material was added to the filling and recycling tank. For the purposes of the experiment, a series of audible alerts were adopted, but clearly, a richer set of alerts and indicators would be feasible via the control device interface. When the control device sent out one long and one short warning tone, it indicated that the upper surface of the solid alloy had reached the upper limit of the set interface level values, at which point, addition of the grouting material was stopped. The control device was used to connect the three-position four-way solenoid valve to Channel (4) (see Table 3) and input the normal-temperature heating medium into the recycling tank. In this case, water was used as the heating medium. When the control device emitted two long warning tones and one short one, it indicated that the liquid level of the heating medium had reached the lower value of the liquid level limits. At this point, the control device started the heater. When the control device sent out two long and two short warning tones, it indicated that the liquid level had reached its upper limit and the recycling device was stopped. The heating medium and alloy grouting material continued to be heated until the control device sent out three long warning tones and one short one, which indicated that the alloy grouting has reached its set temperature above the melting point of the alloy. The preparation of the liquid alloy grouting was then complete and the PID controller was used to control the operation of the heater so as to keep the grouting material at a constant temperature.

When the control device sent out one long and two short warning tones while filling the machine tool foundation with alloy grouting, it indicated that the level of the liquid alloy grouting material in the filling and recycling tank had dropped below the lower limit for the interface level. More solid alloy grouting then needed to be added over time to ensure a continuous supply. When the control device sent out two long and three short warning tones, it indicated that the level of the heating medium in the recycling tank had hit its lower limit. It was then necessary to start the recycling device to supplement the heating medium.

### 4.2. Pouring the Machine Tool Foundation

The main body frame with its four leveling devices was placed over the foundation pit. According to the re-scaled dimensions of an assumed lathe bed, foundation bolt assemblies were fixed on the main body frame and suspended over the foundation pit as described in Section 3.2. With the aid of the leveling bolts and the gradienter installed in the third steel channel, the upper face of the main body frame was made level so that the axis of each foundation bolt was perpendicular to the horizontal plane.

The solidus temperature of Bi-Sn-In alloy grouting material is 80.5 °C. In order to prevent the grouting material from solidifying too quickly, it was necessary to preheat the foundation pit. After starting the filling device and connecting it to Channel (3) (see Table 3), the high-temperature heating medium was injected into the foundation pit. When the liquid level of the heating medium in the foundation pit had reached 1/4 of the depth of the foundation pit, the recycling device was started and Channel (5) was connected. The heating medium, whose temperature was lowered because of the preheating, was pumped into the recycling tank for reheating and re-injected into the foundation pit through Channel (3) of the filling device. This multiple heating and recycling of the heating medium can make the system meet the pouring requirements of large machine tool foundations, despite a limited capacity in the filling and recycling tank. There is, however, some loss of the heating medium over multiple cycles, so, for larger foundations, a reserve supply will need to be in place.

When the liquid level of the heating medium in the foundation pit had risen to 4/5 of the depth of the foundation pit, the control device closed Channel (3) and connected Channel (1). The liquid alloy grouting was then injected into the foundation pit. At the same time, the recycling device continued to extract heating medium through Channel (5) to prevent the foundation pit from overflowing.

With the continuous injection of grouting material, the bent hook part of the foundation bolt assembly was gradually embedded in the machine tool foundation. When the level of the grouting material had risen to a preset height, the control device sent out commands that simultaneously stopped the operation of both the filling device and the recycling device. After this, the temperature of the liquid alloy grouting began to decrease through heat loss and it began to solidify. When the alloy grouting had solidified completely, the temperature dropped to the locally ambient temperature. At this point, the recycling device was started again to recycle the heating medium remaining on the upper surface of the solid alloy grouting material, pumping it back to the filling and recycling tank. Then, the fixing device was disassembled, the main body frame was removed, the construction site was cleaned up, and the construction of the machine tool foundation was completed.

By connecting Channel (2) and Channel (6), the alloy grouting material transportation channels in the filling device and the recycling device were cleaned with the high-temperature heating medium to prevent the creation of blockages.

### 4.3. Recycling of Discarded Grouting Material

After starting the recycling device and connecting Channel (4), heating medium at a normal temperature was imported into the recycling tank. The heating medium was heated by the heater until the temperature reached its set temperature above the melting point of the alloy grouting material.

The main body frame was positioned once more above the previously prepared machine tool foundation.

After connecting Channel (3), the high-temperature heating medium in the filling and recycling tank was injected into the foundation pit to liquefy the LMP alloy.

When the liquefied alloy grouting had reached a specified quantity, Channel (5) was connected and both the liquid alloy grouting and the heating medium were recycled in the filling and recycling tank. During this process, the high-temperature heating medium was continuously injected into the foundation pit through Channel (3) until the solid alloy grouting had fully melted and been completely recycled. When scaled-up, the rate of injection will need to be controlled as only small quantities of the heating medium can be added initially, but this can increase as the volume of removed material increases.

Then, the construction site was cleaned up and the abandoned foundation bolts, steel rounds, etc., were recovered for recycling and/or reuse.

After Channel (6) had been connected, the alloy grouting material transportation channel in the recycling device was flushed through with the high-temperature heating medium.

### 4.4. Results and Discussion

In order to assess the relative performance of the proposed system, a comparison of the LMP alloy-based approach and current approaches based upon the use of polymer concrete was also conducted. Performance assessment was made in relation to three key metrics: construction time; construction cost; and environmental impact. The comparison was undertaken by first pouring LMP alloy grouting, and then polymer concrete (as described by both Suh and Lee [9] and, more recently, by Bedi et al. [21]), into the foundation pit of the prototype. After each pouring, the foundation was dismantled. The overall dimensions of the foundation pit were 800.0 × 600.0 × 600.0 cm^3^. The LMP alloy foundation was constructed in accordance with the specifications stated in Section 3.1, Section 3.2 and Section 3.3 above. The construction of the polymer concrete foundation was carried out in accordance with the standard Chinese code for the construction of concrete structures [22].

#### 4.4.1. Construction Time

The time consumed for constructing and dismantling the two types of machine tool foundation is shown in Table 5, together with the relative efficiency of constructing machine tool foundations with the two kinds of material.

In order to save the test cost of constructing an old machine tool foundation, the time taken to dismantle the foundation is regarded as the time consumed for dismantling the old foundation for the purposes of analysis. It took 13.5 h to construct the machine tool foundation out of the LMP alloy, which is 2% of the time spent in constructing the foundation out of polymer concrete. As can be seen from Table 5, using polymer concrete to build a machine tool foundation requires a lot of concrete maintenance time. When using an LMP alloy, the machine tool can be installed immediately after the LMP alloy has solidified and cooled to room temperature, which saves a considerable amount of time.

#### 4.4.2. Construction Cost

The cost of constructing a machine tool foundation mainly includes the material, water, electricity, manpower, and equipment. The cost of two consecutive constructions was calculated for the two types of machine tool foundation. The results are shown in Table 6.

When constructing the machine tool foundation for the first time, the construction cost of an LMP alloy foundation is 255% that of a polymer concrete foundation because of the relatively high price of the actual LMP alloy grouting material. However, when constructing a new machine tool foundation in the place of a prior foundation (i.e., a second or subsequent construction), the LMP alloy can be recycled and reused and the original foundation pit can also be reused to construct the new foundation. At this point, the construction cost for the LMP alloy is reduced to just 12% of that of a polymer concrete foundation.

#### 4.4.3. Environmental Impact

As a measure of environmental impact, a comparison of the carbon emissions for the two materials was conducted. The carbon emitted during the construction of a machine tool foundation mainly relates to the preparation of the grouting materials, the consumption of water and electricity, and constructor respiration. Standard IPCC (Intergovernmental Panel on Climate Change) emission factor data for the main sources of carbon emissions [23] were used to calculate the carbon emissions involved in constructing a foundation pit. The results are shown in Table 7.

After analyzing the data in Table 7, it could be seen that constructing a machine tool foundation out of an LMP alloy produces carbon emissions of 38 kg, accounting for 23.1% of the carbon emissions produced by constructing a machine tool foundation out of polymer concrete. The experiments show that, compared to the most-recently developed polymer concrete, using an LMP alloy as a grouting material for machine tool foundations is a significantly more environmentally-friendly approach to construction.

By using a prototype version of the system, it was possible to compare the relative time taken to construct a machine tool foundation out of an LMP alloy and out of the most recent forms of polymer concrete. It was also possible to compare the relative economic cost and environmental cost of using the two materials. It was found that using an LMP alloy offers a 98% time saving, an 88% cost saving (for second and subsequent constructions), and a 76.9% reduction in environmental impact. This underscores the potential advantages of adopting this new approach to machine tool foundation construction.

## 5. Conclusions

An LMP alloy machine tool foundation construction system has the potential to replace traditional concrete grouting-based approaches with an LMP alloy. This means that the pouring of machine tool foundations can be accomplished as a single process, significantly shortening the time required for construction. When alloy foundations made by this system have cooled to room temperature, machine tools can be installed on them immediately, without waiting for the typical 15 days required for concrete foundations. An LMP alloy also overcomes concerns such as the need for waterproofing and anti-corrosion treatments.

If it is necessary to remove the LMP alloy machine tool foundation so that a new machine tool can be installed at the same site, the current LMP alloy foundation can be rapidly recycled from the foundation pit and reused to produce the new foundation. This not only reduces the construction costs and shortens the construction period, but also makes it possible to design and construct all-purpose and reusable machine tool foundations that can service a wide range of machine tool foundation needs.

To sum up, the proposed system

1. provides a novel use of new forms of low melting point (LMP) alloy to replace the use of concrete and concrete-polymer hybrids in the construction of machine tool foundations;
2. approaches this in a way which will ensure that machine tool foundations are both structurally flexible and open to rapid recycling;
3. uses an innovative ‘hot bath’ method that makes it possible to directly apply the LMP alloys grouting in situ, without the need for any additional pre-processing, and that facilitates easy recycling of the material; and
4. provides comprehensive coverage of the whole construction and recycling process within a single procedure, thereby speeding up the construction time, significantly improving the construction efficiency, and lowering the construction cost.

## Figures and Tables

**Table 1:** Type, function, and parameters of the sensors.

Name	Type	Function	Parameters
Temperature sensor	Copper-constantan thermocouple	Measuring heating temperature of alloy	−200–350 °C
Grouting material liquid level sensor	Differential pressure liquid level meter with double flanges	Measuring liquid level of alloy	The length of the capillary tube is 1.5 m, which is filled with high-temperature silicone oil. The operating temperature is −20–315 °C
Heating medium liquid level sensor	Differential pressure liquid level meter with single flange	Measuring liquid level of heating medium	The capillary tube is filled with high-temperature silicone oil. The operating temperature is −20–315 °C

**Table 2:** Filling device and recycling device functions.

Name	Functions
Filling device	Fill liquid alloy grouting material
	Clean filling channel
	Fill the heating medium with high-temperature liquid
Recycling device	Fill the heating medium with normal-temperature liquid
	Recycle alloy grouting material and heating medium
	Clean recycling channel

**Table 3:** Material transportation channels.

Associated Process	Channel
Filling liquid alloy grouting material	Channel (1): **liquid alloy grouting material** (filling and recycling tank)→conduit J1→two-position three-way solenoid valve→conduit J2→filling pump→conduit J3→machine tool foundation construction subsystem
Cleaning filling channel	Channel (2): **high-temperature heating medium** (filling and recycling tank)→conduit J4→two-position three-way solenoid valve→conduit J2 →filling pump→conduit J3
Filling the heating medium with high-temperature liquid	Channel (3): **high-temperature heating medium** (filling and recycling tank)→conduit J4→two-position three-way solenoid valve→conduit J2→filling pump→conduit J3→machine tool foundation construction subsystem
Filling the heating medium with normal-temperature liquid	Channel (4): **normal-temperature heating medium** (outside of the system)→conduit H1→three-position four-way solenoid valve→conduit H4→recycling pump→conduit J5→filtering device→filling and recycling tank
Recycling alloy grouting material and heating medium	Channel (5): recycling of **grouting material and heating medium** discarded **liquid alloy grouting material** or **cooled heating material** (machine tool foundation construction subsystem)→conduit H2→three-position four-way solenoid valve→conduit H4→recycling pump→conduit H5→filter device→filling and recycling tank
Cleaning recycling channel	Channel (6): **high-temperature heating medium** (filling and recycling tank)→conduit H3→three-position four-way solenoid valve→conduit H4→recycling pump→conduit H5→filtering device→filling and recycling tank

Note: Items in bold indicate the material transported in the channel.

**Table 4:** Main materials that make up this prototype.

Name	Type	Performance and Parameters
Nano-based soft felt thermal insulating layer	NGEL650A	0.003–0.012 w/(k·m)
Polytetrafluoroethylene (PTFE) film	SFM-3	Operating temperature −200–250 °C Surface tension 18 × 10^−5^ N/cm
Heater	304 stainless steel double-ended U-shaped electric heater pipe	220 V, 1500 W
Filling pump	MD-15FX-220N	135 L/min, 3–265 W, AC100V
Two-position three-way solenoid valve	31A3FV15-U	0–18 bar, −10–140 °C
Recycling pump	MD-15FX-220N	135 L/min, 3–265 W, AC100V
Three-position four-way solenoid valve	2W200-20	0–20 bar, −5–150 °C
Filtering device	SDDX	2–5 m^3^/h
Single-chip microcomputer	STC89C52	5.5–3.3 V, 0–40 MHz

**Table 5:** Machine tool foundation construction time.

Construction material	Construction Procedure		Time Consumed (h)	Total Time Consumed (h)
LMP alloy	Preparing LMP alloy grouting material		2.0	13.5
	Constructing machine tool foundation	Fixing foundation bolts; Leveling main framework	1.0	
		Preheating foundation pit	0.5	
		Injecting LMP alloy grouting material	2.0	
		Forming machine tool foundation and cooling it to room temperature	4.0	
	Dismantling machine tool foundation	Heating heating medium	1.0	
		Liquefying the solid-state LMP alloy in the foundation pit	2.0	
		Recycling the LMP alloy	1.0	
Polymer concrete	Preparing polymer concrete grouting material		1.5	692.5
	Constructing machine tool foundation	Pouring machine tool foundation	3.0	
		Maintaining polymer concrete	24.0 × 14.0 = 336.0	
		First grouting	2.0	
		Maintaining polymer concrete	24.0 × 7.0 = 168.0	
		Second grouting	2.0	
		Maintaining polymer concrete	24.0 × 7.0 = 168.0	
	Dismantling machine tool foundation		12.0	

**Table 6:** Machine tool foundation construction cost.

Construction Iteration	Construction Material	Construction Procedure	Construction Cost (KCNY)	Total Cost (KCNY)
First construction	LMP alloy	Constructing foundation pit	5.0	102.0
		Preparing LMP alloy grouting material	95.0	
		Constructing machine tool foundation of LMP alloy	2.0	
First construction	Polymer concrete	Constructing foundation pit	5.0	40.0
		Preparing polymer concrete grouting material	5.0	
		Constructing machine tool foundation of polymer concrete	30.0	
Second construction	LMP alloy	Dismantling machine tool foundation of LMP alloy	2.0	5.0
		Preparing LMP alloy grouting material	1.0	
		Constructing machine tool foundation of LMP alloy	2.0	
Second construction	Polymer concrete	Dismantling machine tool foundation of polymer concrete	2.0	42.0
		Constructing foundation pit	5.0	
		Preparing polymer concrete grouting material	5.0	
		Constructing machine tool foundation of polymer concrete	30.0	

**Table 7:** Carbon emitted during the construction of a machine tool foundation.

Machine Tool Foundation Type	Main Carbon Emission Sources	Carbon Emission Factor	Carbon Emission (kg)	Total Carbon Emissions (kg)
LMP alloy	Preparing LMP alloy grouting material	116 kg CO_2_/m^3^	23.2	38.0
	Electricity	0.785kg CO_2_/kwh	11.8	
	Water	0.26 kg CO_2_/m^3^	0.1	
	Constructor respiration	0.73 kg CO_2_/d	2.9	
Polymer concrete	Preparing polymer concrete grouting material	290 kg CO_2_/m^3^	58.0	164.7
	Electricity	0.785 kg CO_2_/kwh	23.6	
	Water	0.26 kg CO_2_/m^3^	1.3	
	Constructor respiration	0.73 kg CO_2_/d	81.8	
